# Supplementary material for: Mechanisms governing the pioneering and redistribution capabilities of the non-classical pioneer PU.1
Source: Nat Commun. 2020 Jan 21;11:402. doi: 10.1038/s41467-019-13960-2 (PMC6972792; doi:10.1038/s41467-019-13960-2)
Supplement: Supplementary file 7 — Source data [file 41467_2019_13960_MOESM7_ESM.zip › Source_Data/Figure6/Figure6B_MotifScanOutput/ETS1_PU.1/homerResults/motif52.similar.html]

motif52

## Information for motif52

T
C
G
A
T
C
A
G
G
C
A
T
A
C
G
T
G
A
T
C
G
A
C
T
G
A
T
C
T
C
G
A
A
G
T
C
T
C
A
G
T
C
G
A
A
C
T
G
T
C
G
A
  
Reverse Opposite:  

A
C
G
T
T
G
A
C
A
G
C
T
A
G
T
C
T
C
A
G
A
G
C
T
C
T
A
G
C
T
G
A
C
A
T
G
T
G
C
A
C
G
T
A
A
G
T
C
A
G
C
T
  

|  |  |
| --- | --- |
| p-value: | 1e-19 |
| log p-value: | -4.507e+01 |
| Information Content per bp: | 1.695 |
| Number of Target Sequences with motif | 199.0 |
| Percentage of Target Sequences with motif | 1.26% |
| Number of Background Sequences with motif | 150.7 |
| Percentage of Background Sequences with motif | 0.47% |
| Average Position of motif in Targets | 103.7 +/- 60.7bp |
| Average Position of motif in Background | 86.6 +/- 63.5bp |
| Strand Bias (log2 ratio + to - strand density) | 0.1 |
| Multiplicity (# of sites on avg that occur together) | 1.02 |
| Motif File: | file (matrix) reverse opposite |

### Similar de novo motifs found

|  |  |  |  |  |  |  |  |
| --- | --- | --- | --- | --- | --- | --- | --- |
| Rank | Match Score | Redundant Motif | P-value | log P-value | % of Targets | % of Background | Motif file |
| 1 | 0.958 | C A G T T G A C G A C T A G T C T C G A A G C T T C A G C T G A T C A G C G T A C G T A G T A C G C A T A T C G | 1e-17 | -39.566137 | 1.00% | 0.35% | motif file (matrix) |
| 2 | 0.943 | A G C T G T A C A G C T A G T C C T A G G A T C C T A G C T G A A C T G G T C A C G A T A G T C | 1e-16 | -37.292171 | 1.16% | 0.46% | motif file (matrix) |
| 3 | 0.885 | G T C A A C G T T G A C G A C T G A T C T C A G A G T C A T C G C T G A A C T G T G C A | 1e-15 | -35.147446 | 1.11% | 0.45% | motif file (matrix) |
